# Supplementary material for: Programmable DNA looping using engineered bivalent dCas9 complexes
Source: Nat Commun. 2017 Nov 20;8:1628. doi: 10.1038/s41467-017-01873-x (PMC5696343; doi:10.1038/s41467-017-01873-x)
Supplement: Supplementary file 1 — Supplementary Information [file 41467_2017_1873_MOESM1_ESM.pdf]

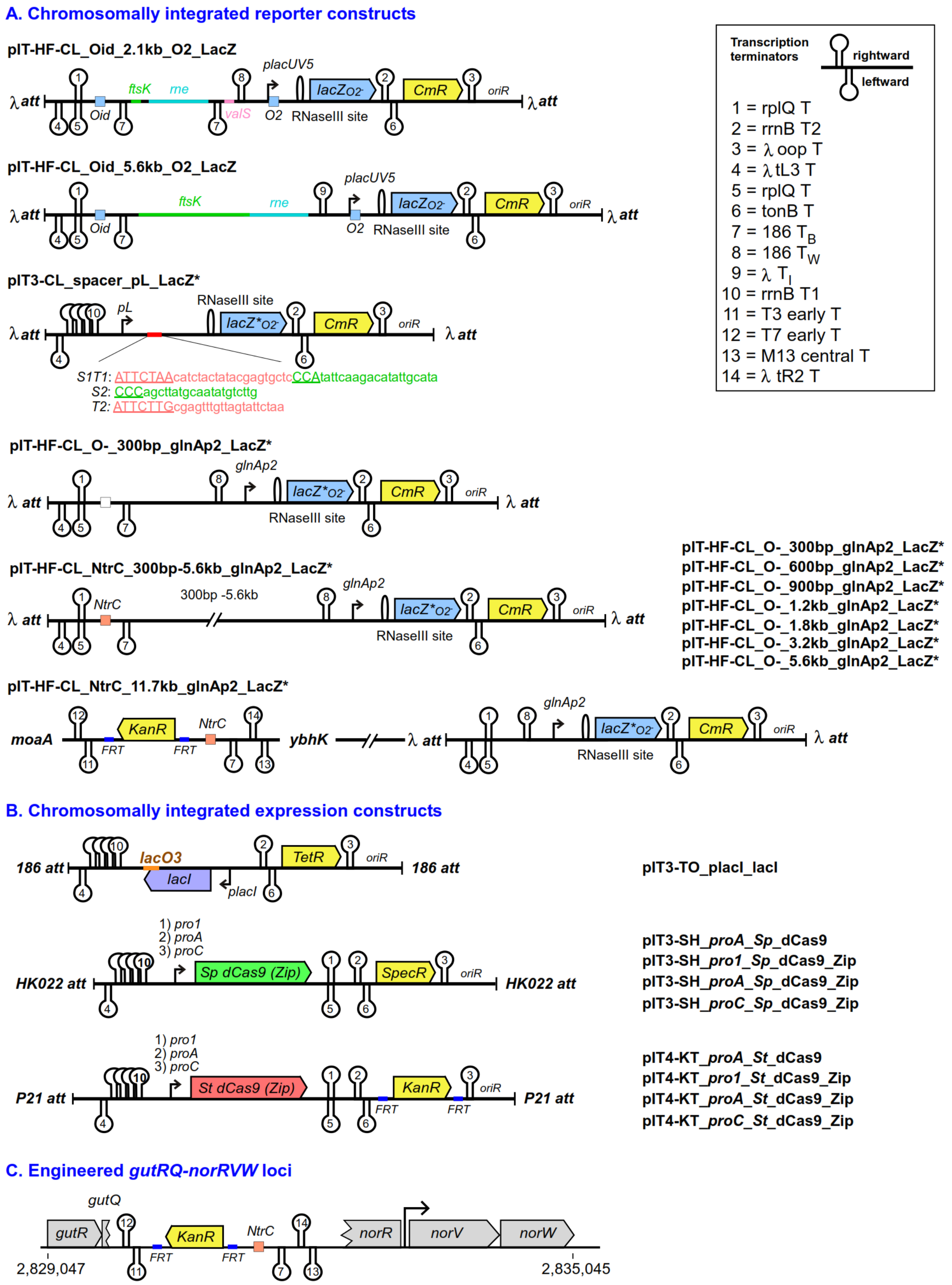

Supplementary Figure 1. Details of chromosomally integrated constructs.

A. LacZ reporter constructs. LacZ* refers to *lacZ* gene with a modified (weaker) ribosome binding site. *NtrC* indicates the NtrC enhancer module (*glnA* sites 1 and 2).
B. LacI and dCas9 expression constructs.
C. NtrC enhancer module placed upstream of the *norRVW* locus.

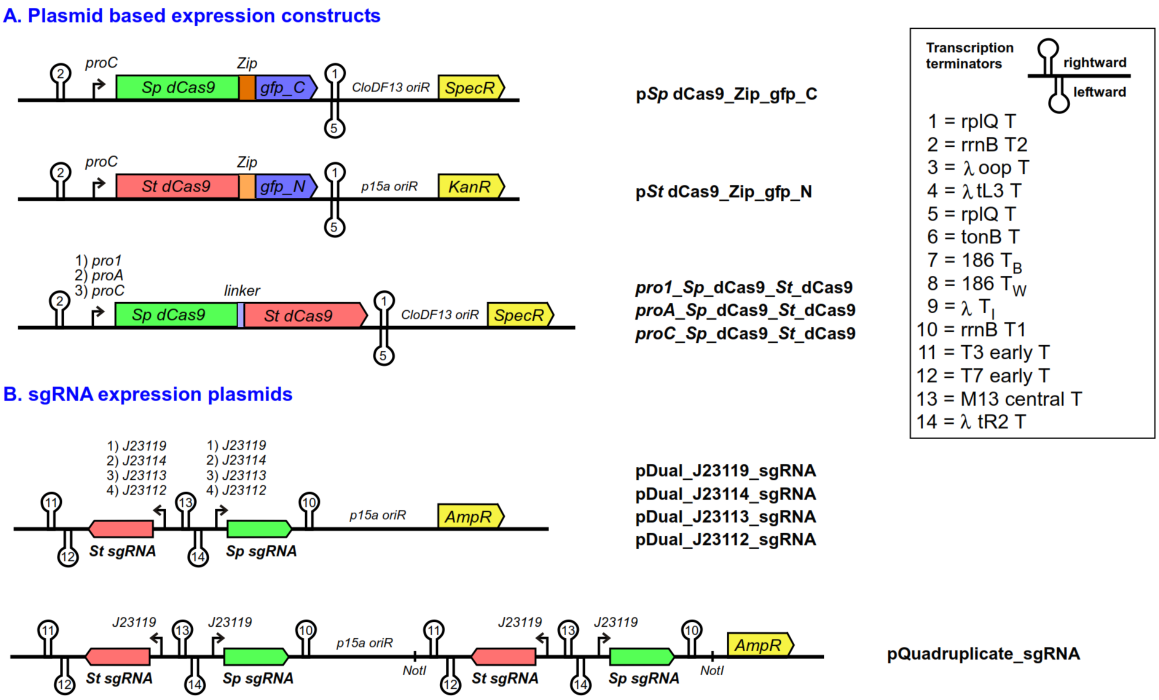

Supplementary Figure 2. Details of plasmid based expression constructs.

A. dCas9 expression plasmids.
B. Guide RNA expression constructs.

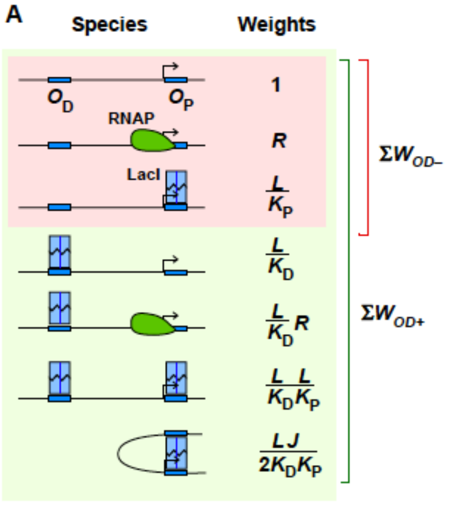

L = [LacI] (nM)

R = weight for RNAP occupation (unitless)

K_P = dissociation constant for LacI at O_P (nM)

K_D = dissociation constant for LacI at O_D (nM)

J = DNA looping factor (nM)

Fraction looped (OD+): $F = \dfrac{LJ/2K_DK_P}{\Sigma W_{OD+}}$

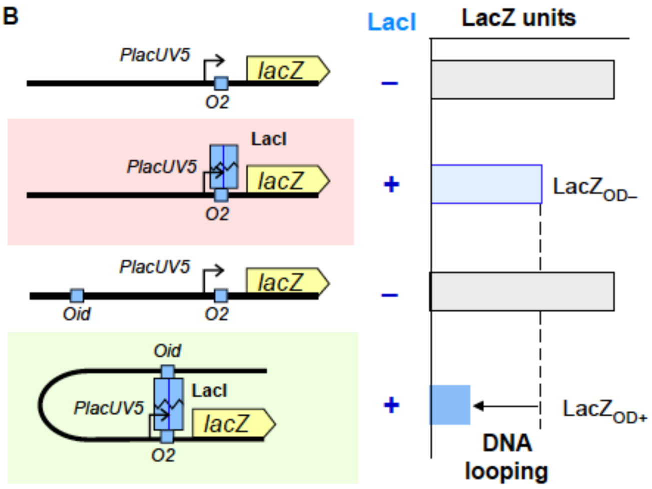

LacZ units in absence or presence of O_D:

$$\mathrm{LacZ}_{OD-} = a\left(\frac{R}{\Sigma W_{OD-}}\right) - bkgd$$

$$\mathrm{LacZ}_{OD+} = a\left(\frac{R+RL/K_D}{\Sigma W_{OD+}}\right) - bkgd$$

a = factor relating promoter occupation by RNAP to LacZ units

$bkgd$ = unrepressible background LacZ units

C

$$\frac{(\mathrm{LacZ}_{OD-}-bkgd)-(\mathrm{LacZ}_{OD+}-bkgd)}{(\mathrm{LacZ}_{OD-}-bkgd)} = \frac{\dfrac{R}{\Sigma W_{OD-}} - \dfrac{R+RL/K_D}{\Sigma W_{OD+}}}{\dfrac{R}{\Sigma W_{OD-}}} = 1 - \frac{(1+L/K_D)\,\Sigma W_{OD-}}{\Sigma W_{OD+}}$$

$$= 1 - \frac{(1+L/K_D)(1+R+L/K_P)}{\Sigma W_{OD+}} = 1 - \frac{1+R+L/K_P+L/K_D+RL/K_D+L^2/K_PK_D}{\Sigma W_{OD+}} = \frac{LJ/2K_PK_D}{\Sigma W_{OD+}} = F$$

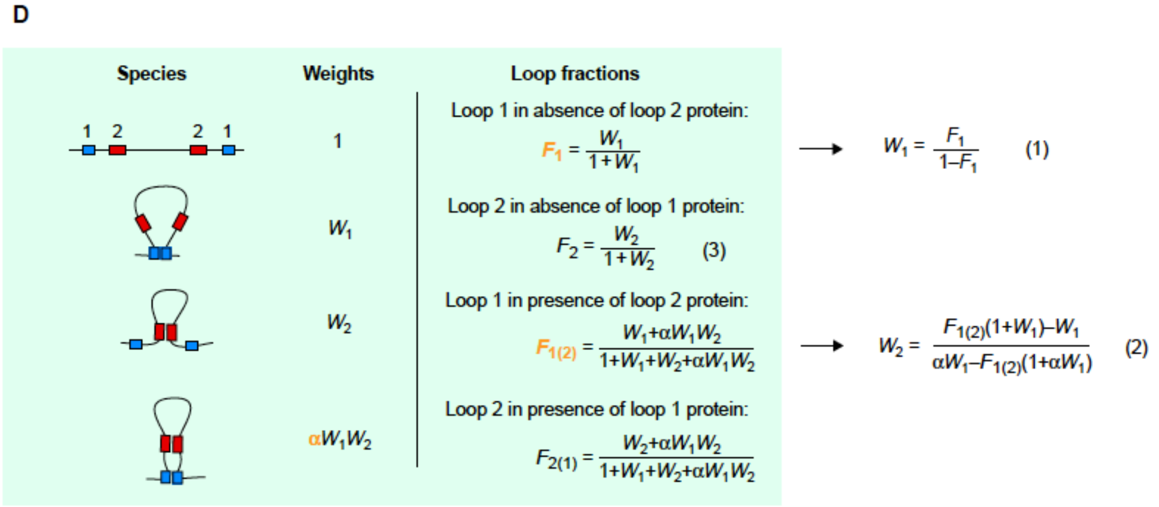

Supplementary Figure 3. Loop modelling.

A. Statistical-mechanical model for LacI repression with two operators and DNA looping [1]. Shaded boxes indicate species available in the absence or presence of the distal operator.

B. LacI-mediated DNA looping in the presence of the distal operator can improve repression of the promoter. Just one of the species available in each condition is shown (see A). The equations show how the observed LacZ units can be related to the model parameters in A.

C. Interconversion of the observed LacZ units ± OD and the fractional looping, F, using equations from A and B.

D. A statistical-mechanical two-loop model [2] allows calculation of internal loop formation from: (1) measurement of the fractional looping of the external loop in the absence of the internal loop, F_1 (measured for the LacI loop without dCas9); (2) measurement of the fractional looping of the external loop in the presence of the internal loop, $F_{1(2)}$ (measured for the LacI loop with dCas9); and (3) the loop assistance factor, α, which quantitates how much the formation of one loop favours the formation of the other. For a 2.1 kb LacI loop with a 1.4 kb internal loop formed by λ CI, this assistance factor was ~3 [2], roughly equivalent to the fractional change in the effective length of DNA looped by LacI in the absence of CI looping (2.1 kb) compared to in its presence (2.1-1.4 = 0.7 kb). Thus, we expect α ~ 2100 bp/(300 bp+390 bp) ~ 3 for the reporters in Figure 4B, and α = 5600 bp/(300 bp+590 bp) ~ 6.2 for the reporters in Figure 5A. F_1 allows calculation of W_1 (eqn 1). This and the other values allows calculation of W_2 (eqn 2). F_2, the fractional looping of the internal (dCas9) loop in the absence of the external (LacI) loop can be calculated from W_2 (eqn 3).

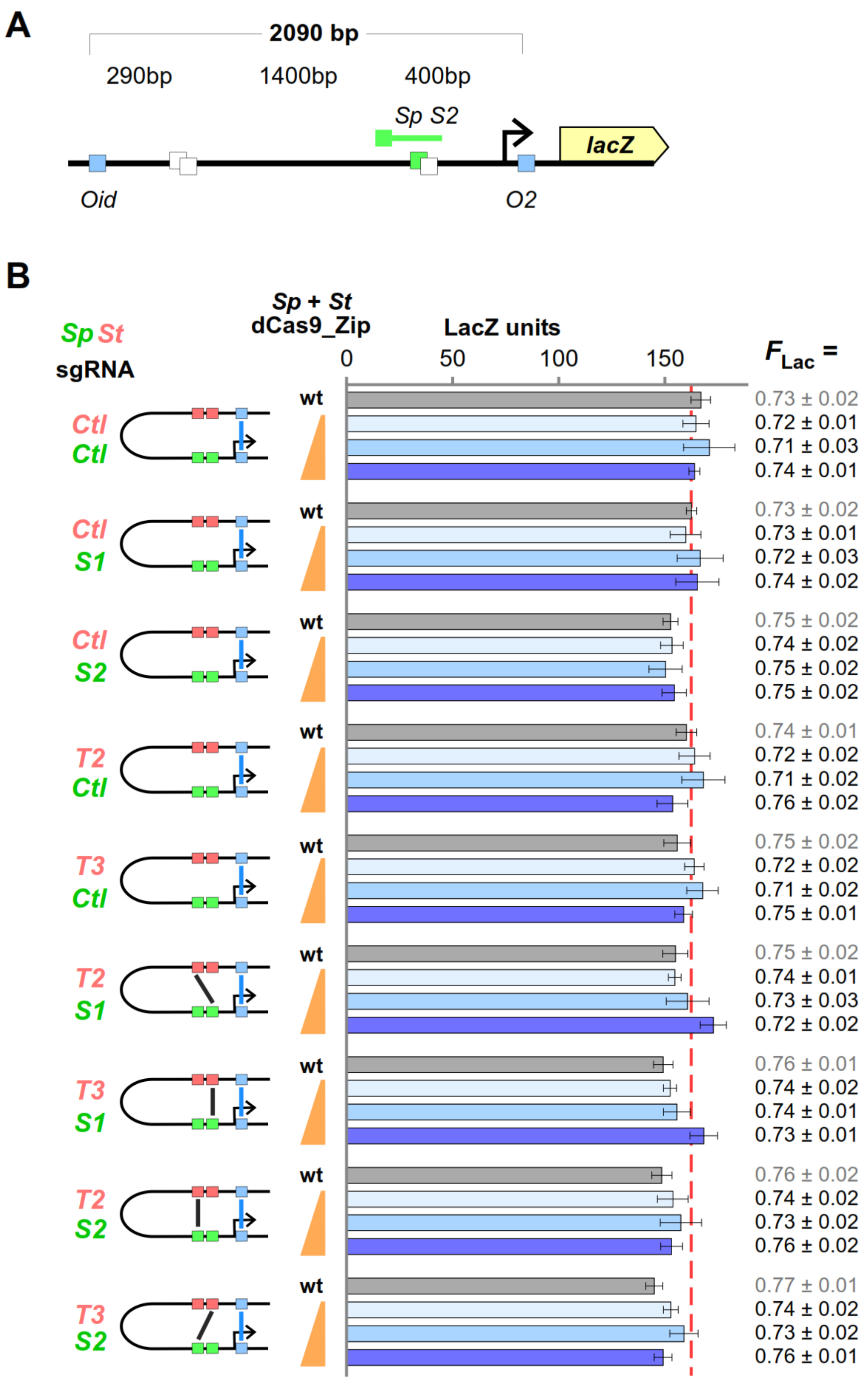

Supplementary Figure 4. Binding of bivalent dCas9 to its target DNA is required for dCas9 mediated DNA looping.

A. Schematic representation of the 1.2 kb looping reporter with all but one dCas9 target site removed.

B. Lack of DNA looping by bivalent dCas9s in the absence of specific dCas9 target sites. Data are mean ± 95% confidence intervals (n = 9). F_{loop} is calculated as [(LacZ$_{OD-}$–*bkgd*) – (LacZ$_{OD+}$–*bkgd*)] / (LacZ$_{OD-}$–*bkgd*) (Supplementary Figures 3A-C), and is expressed as mean ± standard deviation (n = 9).

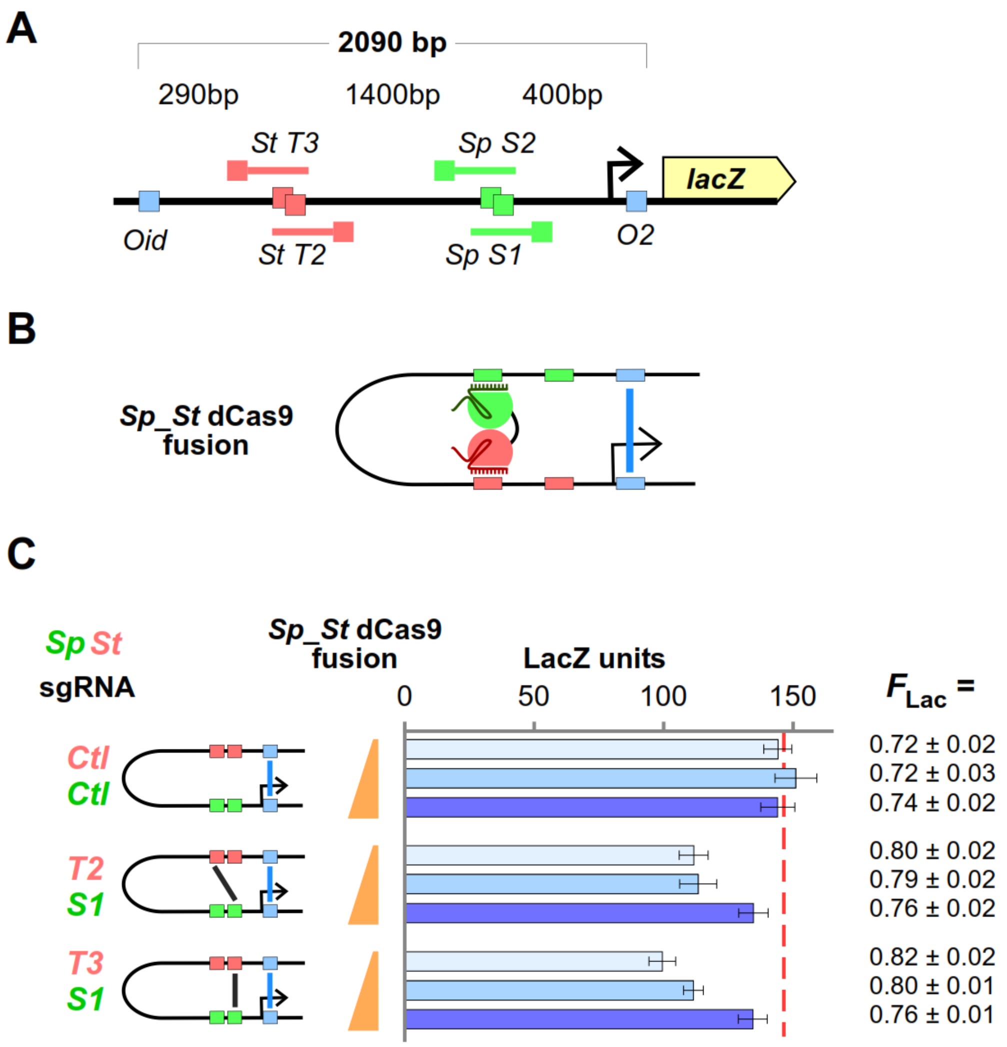

Supplementary Figure 5. DNA looping via *Sp_St*_dCas9 fusion.

A. Schematic representation of the 1.2 kb looping reporter (see also Supplementary Figure 1).

B. A cartoon representation of a nested loop formed by the *Sp_St*_dCas9 fusion complex and the Lac repressor.

C. The formation of CRISPR loop via *Sp_St*_dCas9 fusion assists the formation of the LacI looping. Data are mean ± 95% confidence interval (n = 9). F_{loop} values are mean ± standard deviation (n = 9).

Supplemental Table 1. List of spacer sequences used in this study.

	Spacer sequences (5' to 3')
Sp (*St*) *Ctl* sgRNA	AACTTTCAGTTTAGCGGTCT
Sp sgRNA *S1*	TATGCAATATGTCTTGAATA
Sp sgRNA *S2*	CAAGACATATTGCATAAGCT
Sp sgRNA *S3*	ATTCGCGGTTTTCGACTTCC
Sp sgRNA *S4*	GGGTTCAGTTAGTCACCTGC
Sp sgRNA *S5*	TCCCTCTCAAGCCGCCAGCA
Sp sgRNA *S6*	TCTGAGACGTGATGGTGGCG
Sp sgRNA *S7*	ACCATTCCCGTCATTATTGT
Sp sgRNA *S8*	TTGCTGAAAAACTCGGCGGC
St sgRNA *T1*	GAGCACTCGTATAGTAGATG
St sgRNA *T2*	TTAGAATACTAACAAACTCG
St sgRNA *T3*	ATTCTAACATCTACTAGAAT
St sgRNA *T4*	GTTTAAGGTCCCGTGACAAG
St sgRNA *T5*	CGGTAGTCGCACCGGTGGTT
St sgRNA *T6*	CTGAAGGCAGCGATAATCGC
St sgRNA *T7*	CTGATTGAACAACTGGAAAG

Supplemental Table 2. List of primers used in chromosome conformation capture assays.

Primer Name	Primer sequence
P1	5’ TGCTCGTAACGCACTTTCTG 3’
P2	5’ GCAGATACACTTGCTGATGCG 3’
P3	5’ GGACAAACTCAAGGTCATTCGC 3’
P4	5’ ACGTTAGATACCCAGCTTATGC 3’
P5	5’ CCGAAATGGTTGCCGATGTG 3’

Supplemental Table 3. List of primers used in qRT-PCR experiments.

Target Name	Primer sequence	Primer Efficiency	Amplicon size
Sp Cas9	5’ ATGGAGAGATTCGCAAACGC 3’	94.8%	94bp
	5’ TTGCGCACTGTGGCAAAATC 3’		
St Cas9	5’ ACATCCGCAAGTACAGCAAG 3’	93.8%	118bp
	5’ ACCACCTTGTTGTTGCTGTC 3’		
norV	5’ TGGCACAAATTCCCGATACG 3’	94.6%	143bp
	5’ AATGAGCTGTTTGCCGTTGC 3’		
norW	5’ TGCACCTGTTTCCACAAACC 3’	100.2%	91bp
	5’ AGCTTGTCGTATTGCCACTG 3’		
gyrA	5’ TGGAAGTTGACGCCAAAACC 3’	94.9%	124bp
	5’ ATGCCTTCCACGCGTTTTTC 3’		
rho	5’ AAATCCGCCGTTTCAACCTC 3’	89.7%	137bp
	5’ TTTGTTGCGGGCGTTTTCAG 3’		

Supplementary References

1. Priest DG, Cui L, Kumar S, Dunlap DD, Dodd IB, Shearwin KE. Quantitation of the DNA tethering effect in long-range DNA looping in vivo and in vitro using the Lac and lambda repressors. *Proc Natl Acad Sci U S A* **111**, 349-354 (2014).

2. Priest DG, Kumar S, Yan Y, Dunlap DD, Dodd IB, Shearwin KE. Quantitation of interactions between two DNA loops demonstrates loop domain insulation in E. coli cells. *Proc Natl Acad Sci U S A* **111**, E4449-4457 (2014).
